# Theoretical Prediction of Core-Level Binding Energies: Analysis of Unexpected Errors

**DOI:** 10.1021/acs.jpca.3c07567

**Published:** 2024-01-25

**Authors:** Carmen Sousa, Paul S. Bagus, Francesc Illas

**Affiliations:** † Departament de Ciència de Materials i Química Física & Institut de Química Teòrica i Computacional (IQTCUB), Universitat de Barcelona, C/Martí i Franquès 1, Barcelona 08028, Spain; ‡ Department of Chemistry, University of North Texas, Denton, Texas 76203-5017, United States

## Abstract

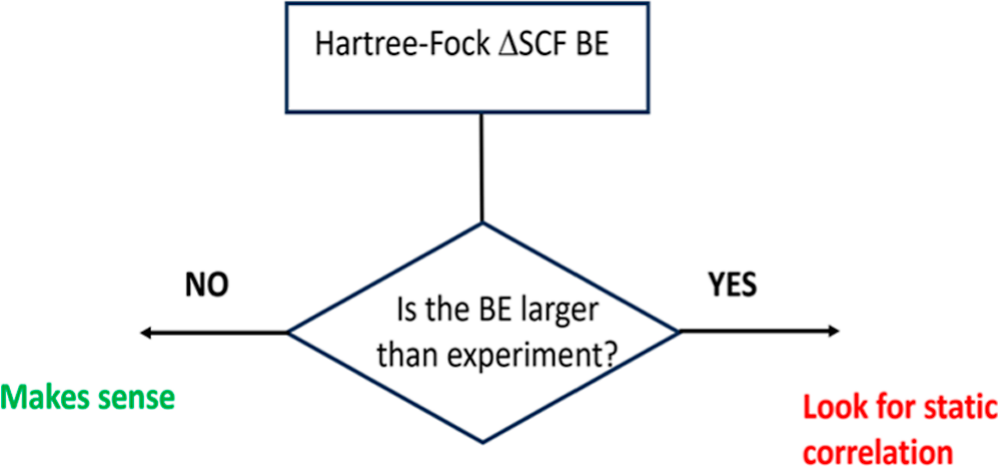

The analysis of the C(1s) and O(1s) core-level binding energies (CLBEs) of selected molecules computed by means of total energy Hartree–Fock (ΔSCF-HF) differences shows that in some cases, the calculated values for the C(1s) are larger than the experiment, which is unexpected. The origin of these unexpected errors of the Hartree–Fock ΔSCF BEs is shown to arise from static, nondynamical, electron correlation effects which are larger for the ion than for the neutral system. Once these static correlation effects are included by using complete active space self-consistent field (CASSCF) wave functions that include internal correlation terms, the resulting ΔSCF BEs are, as expected, smaller than measured values.

## Introduction

Since the pioneering work of Siegbahn and co-workers on the development of X-ray photoelectron spectroscopy (XPS),^1,2^ XPS has been widely used to explore the composition and the electronic structure of molecules as well as bulk materials and their surfaces.^3−5^ In XPS, the sample of interest is irradiated by an X-ray of known energy, leading to ionization of a core electron whose kinetic energy is measured. Following the classic photoelectron effect proposed by Einstein, the electron binding energy, BE, is given by BE = *h*ν – KE, where KE is the kinetic energy of the ionized electron. This simple energy balance allows one to obtain the binding energy of core electrons, which are characteristic of each chemical element. In the case of gas-phase molecules, the experiment provides absolute values of the core-level binding energies (CLBEs), whereas for surfaces and solids, absolute CLBEs are harder to obtain as one must account for the work function in the measurement of the kinetic energy.^6^

For a given chemical element, the difference between the CLBEs for that element in different environments, usually referred to as CLBE shifts or ΔCLBE, is small enough to allow the BEs to identify the element ionized but large enough to provide valuable information about the chemical environment of the ionized atom.^7^ Thus, apart from providing information about the elemental composition of a sample, the XPS BEs provide information about the electronic structure, including the oxidation state, of the core ionized atom in the compound studied. The information extracted from ΔCLBEs goes well beyond determining oxidation states, as discussed at length in various papers analyzing, in detail, the origin of the physical mechanisms that govern these shifts.^7−9^ Here, ab initio calculations are invaluable because they allow one to differentiate between initial- and final-state effects. Note also that the Auger effect allows one to separate initial- and final-state effects purely from measured quantities.^10−12^ One must keep in mind that the CLBEs provide information on the ionized materials, whereas one is often interested in the neutral sample. Here is where the distinction between initial and final states becomes crucial, as it indicates whether the observed shift is already present in the unionized material. For additional information about the concepts of the initial and final states and their role in the ΔCLBEs, the interested reader is referred to the pertinent literature.^7−9^

Experimental values of CLBEs for a large number of gas-phase molecules are available in the literature,^13,14^ which prompted theoretical studies to assess the performance of different approaches, as discussed below. The most straightforward way to estimate the CLBEs of a given molecule is the difference in total energy of the neutral molecule and the molecule with a core hole, both computed by the same theoretical method. In a nonrelativistic framework, both energies are ideally obtained from a variational method, as in the self-consistent field (SCF) approach to the Hartree–Fock (HF) energy; the resulting procedure is usually referred to as ΔSCF. While the original studies of XPS were made using nonrelativistic wave functions, this does not allow one to obtain accurate BEs and it does not allow one to determine the spin–orbit splittings of core–shells that have . These deficiencies of nonrelativistic calculations are corrected through the use of Dirac HF and configuration interaction wave functions, as shown, for instance, for the Fe(2p) core in Fe_2_O_3_.^15^ In any case, the ΔSCF procedure is also applicable to density functional theory (DFT)-based methods since the Kohn–Sham equations are usually solved through a SCF approach. While the SCF-HF energy of a neutral molecule can be easily obtained using a large enough basis set and either the experimental or computationally optimized molecular structure, obtaining the energy of the core ionized molecule requires special procedures to avoid the variational collapse to the lowest energy of the corresponding cation. The seminal work of Bagus back in 1963 paved the way to estimate CLBEs from ΔSCF calculations,^16^ showing, in addition, that the thus-calculated CLBEs for Ne-like and Ar-like ions are very close to experiment. A more extensive survey has been published recently,^17^ as discussed below. Here, we need to note that the ΔSCF-HF calculated CLBEs are expected to be smaller than the experimental value, as pointed out by Mulliken.^18^ The reason that the HF ΔSCF BEs are expected to be smaller is that the electron correlation is larger in the neutral molecule than in the ion since the ion has fewer electron pairs; this is a central issue that will be further discussed. In principle, DFT-based methods include electron correlation and thus are supposed to provide more accurate numerical results. Pueyo Bellafont et al. investigated the performance of several density functionals in predicting a total of 185 1s CLBEs of main-group elements of a set of 68 molecules with different functional groups.^17^ These authors compared the performance of HF, PBE, and TPSS, the latter two being representative of generalized gradient approach (GGA) and *meta*-GGA functionals, respectively, corresponding to the third and fourth layers of the so-called Jacob’s ladder. The mean average error (MAE) for HF and TPSS was similar, 0.44 and 0.33 eV, respectively, while that of PBE was significantly larger (1.03 eV). The contribution of relativistic effects was also considered, as estimated from the gas-phase atom, and found to slightly decrease the MAE.

The ΔSCF approaches discussed so far involve two variational calculations, which may face problems when dealing with periodic models due to the use of a charged unit cell for the calculation involving the core-hole. The charge can be neutralized by using a uniform background, although whether this is a realistic representation remains an open issue. Methods based on many-body perturbation theories such as those relying on the GW approach are especially attractive as they do not need to consider a charged unit cell. Following earlier work on the application of Green’ functions to study XPS features,^19,20^ the performance of GW methods on predicting CLBEs of gas-phase molecules has been explored by several authors^21,22^ with results that depend very much on the initial guess density and on the level at which the GW equations are solved, the most accurate ones leading to MAE values with respect to experiments below 0.2 eV, although at a considerable computing cost. Again, the inclusion of relativistic effects slightly improves the results for light atoms, but these may be very large for heavier atoms. Note, however, that GW methods are nonvariational, implying that calculated values can be above or below the exact value. Within this methodology, the goal is to accurately predict CLBEs rather than identify the physical ingredients of the final results.

The preceding discussion regarding the performance of different approaches in predicting CLBEs has focused on statistical analysis. A more detailed inspection of the individual values offers some interesting information. As mentioned, the ΔSCF-HF predicted CLBEs are expected to be smaller than the experimental values, and this is usually the case.^17,23,24^ However, there are cases where this does not hold, meaning that electron correlation needs to be explicitly taken into account. The cases of the lowest ^2^S states of Ne^+^ and Ar^+^ and their isoelectronic ions are well documented, where the effect of the static correlation involves the same shell as the ionized electron.^16^ A similar situation has been found for the CO molecule, where the ΔSCF-HF predicted value for the O(1s) is lower than experiment by ∼0.6 eV, as expected, while the C(1s) CLBE is larger than experiment by ∼1 eV. This would imply that for the C(1s) core hole-state, the contribution of electron correlation is larger than on the initial state, even if the latter has one more electron. Including nondynamical electron correlation through a complete active space self-consistent field (CASSCF) wave function where the 1π and 2π orbitals define the active space leads to values both closer to and smaller than experiment.^25^ The effect of nondynamical electron correlation can be understood as a reorganization of the relative weight of the different covalent and ionic valence bond (VB) forms as the HF wave function tends to assign the same weight to all valence bond forms.^26^ This is precisely the reason beyond the incorrect dissociation limit of the restricted HF (RHF) potential energy curve of the hydrogen molecule. At the equilibrium distance, covalent and ionic resonant forms may have a noticeable contribution, but the ionic forms need to decrease when the internuclear distance increases, and by construction, this is not possible when relying on a RHF description. This incorrect behavior becomes evident in molecules involving multiple bonds such as CO. Including nondynamical electron correlation effects by means of an appropriate CASSCF wave function restores the proper balance between covalent and ionic resonant forms. Note in passing that this also provides a case study to investigate whether a given density functional appropriately introduces the physically meaningful nondynamic electron correlation effects, as discussed in previous work.^27^

The unexpected behavior for the BEs of CO can be better and more informatively understood within the context of molecular orbital, MO, theory, as proposed and pioneered by Mulliken. It requires only the use of the equivalent core model as proposed by Jolly and Hendrickson,^28−30^ where the core ionized atom is replaced by the next atom in the periodic table. Thus, for CO with a C(1s) ionization, the *Z* + 1 model is NO^+^, and for the O(1s) ionization, the *Z* + 1 model is CF^+^. Thus, we must understand why the static correlation effects are different for these three molecules. To be clear, we consider the excitations within the 2s and 2p atomic shells described by Sinanoğlu^31,32^ as internal excitations. The key thing to note is the charge separation of the two atoms in these three diatomic molecules. The separation is largest for CF^+^ where C has *Z* = 6 and F has *Z* = 9 for a difference of 3. It is smallest for NO^+^ where the difference is only one and intermediate for CO where the difference is 2. Now, it is reasonably obvious that the importance of the internal configuration interaction (CI) or multiconfigurational SCF, which gives the static correlation effects, is directly correlated with the degree of charge separation. The static correlation is smaller when the charge separation is larger since the MOs will be more nearly localized on the separate atoms. On the other hand, it will be larger when the charge separation is smaller. This can be seen for the limiting case of a homopolar diatomic molecule where the MOs all have exactly equal contributions from each atom and are either gerade, g, or ungerade, u, orbitals. Thus, the static correlation will be largest for NO^+^, intermediate for CO, and smallest for CF^+^. This immediately shows us that the HF ΔSCF BE will be smaller than experiment for the O(1s) ion since correlation effects are larger for CO than for CF^+^, the equivalent core molecule for the O(1s) hole. On the other hand, correlation effects will be larger for the NO^+^, the equivalent core ion for the C(1s) hole, than for CO. This is consistent with a smaller BE error being smaller than experiment or a ΔSCF BE being larger than experiment, which is precisely what is found for the core level BEs of CO. Thus, the simple application of MO theory, which is not possible with VB theory, shows us when to expect the unusual case where the ΔSCF BEs will be larger than experiment. Clearly, this kind of analysis is applicable in general, as we show in the present paper.

From the preceding discussion, it is clear that the case of CO is likely not to be unique. In fact, a detailed scrutiny of the individual results in the Supporting Information of ref (17) reveals various cases where several ΔSCF-HF predicted CLBEs are larger than experiment, indicating the presence of stronger nondynamic correlation effects in the ionized atom, exactly as discussed in the preceding paragraph. In the present work, we analyze a set of these cases and prove that once nondynamical correlation is included, the calculated CLBEs are smaller than experiment, as expected, and also more accurate. The fact that, in these cases, nondynamical correlation plays such an important role provides a way to check not only the accuracy of density functionals, where the static correlation effects are not explicitly included, but also to verify that the main physics is included.

## Theoretical Methodology and Computational Details

In the present work, we use HF and CASSCF wave functions to analyze the C(1s) and O(1s) CLBEs of a series of molecules with the aim to investigate whether the clear nondynamical electron correlation contribution described for CO is a particular case or rather quite general. The molecules described below have been chosen because some of the ΔSCF-HF calculated CLBEs are larger than experiment. These include the linear CO_2_, CS_2_ and COS molecules featuring two double bonds and four organic compounds, three showing different types of C–O bonds and one with a C–F bond. These are formaldehyde (H_2_CO), formic acid (HCOOH), and methanol (H_3_COH), involving a carbonyl group, a carboxylic group, and a hydroxyl group, respectively, and fluoromethane (CH_3_F), where the C atom is bonded to a highly electronegative atom such as F. In particular, the C(1s) and O(1s) core-level BEs have been computed for all molecules, except for CS_2_ for which the S(2s) CLBEs have been calculated as this is a core level where experimental values are available and F(1s) for CH_3_F.

**Figure 1:**
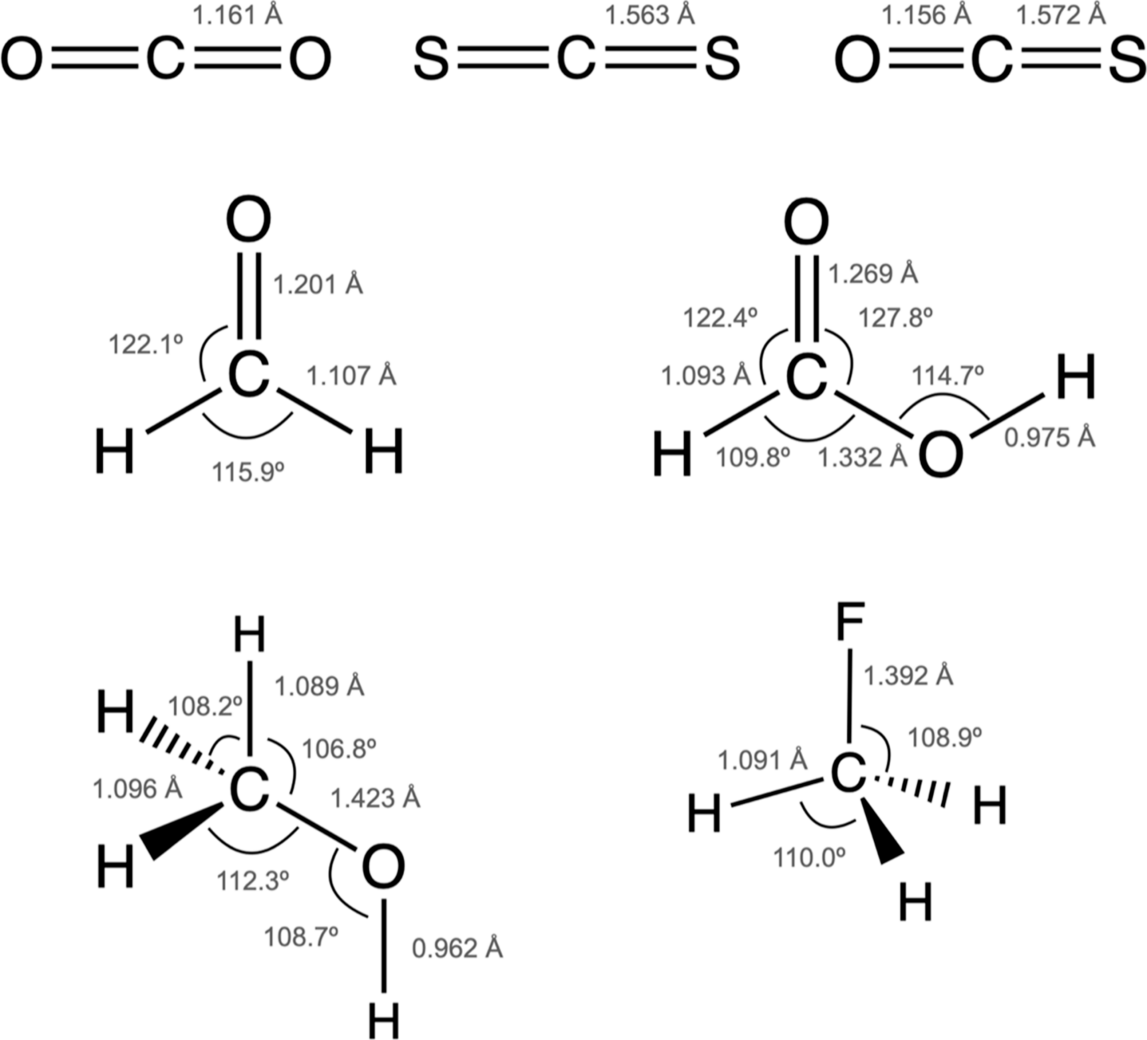
Optimized molecular geometries of the molecules studied.

The HF and CASSCF calculations of the singlet ground state and core-ionized state (doublet) have been carried out at the molecular structure optimized by density functional theory (DFT) calculations applying the hybrid B3LYP functional with a basis set derived from the Ahlrichs valence triple-ζ plus polarization basis set.^33^ Essentially, this implies using the fully uncontracted primitive sets; this is (10s, 6p, 1d) for C, O, and F, (12s, 9p, 1d) for S, where the d functions have five components, and (5s, 1p) for H. The optimized molecular geometries are shown in Figure 1. The HF calculations of the ground and ionized states have been carried out using the GAMESS-06 code,^34,35^ which allows us to ensure the convergence to the core hole state by using the overlap instead of the Aufbau criteria to select the occupied orbitals through the SCF procedure. The importance of dynamic electron correlation can be estimated from the difference between the CASSCF and experimental values, although this difference also accounts for relativistic effects. For completeness, complete active space second-order perturbation theory (CASPT2) values are included for comparison. CASPT2 calculations have been performed using the ionization potential-electron affinity (IPEA) shifts of 0.0 and 0.25 au.^36^ In both cases, the results are very similar, and only the values without IPEA shift are reported in the tables. The CASSCF and CASPT2 calculations have been carried out using the OpenMolcas package.^37^ To converge to the proper hole state, a procedure involving several steps of freezing a subset of the molecular orbitals while the remaining orbitals are varied is required. In the CASPT2 calculations, all electrons except the deep-core 1s^2^ of S are included in the perturbational treatment of the remaining electron correlation.

For the CO_2_, CS_2_, and COS molecules, an active space containing 8 electrons and the 6 orbitals of π character, the occupied 1π_u_ and 1π_g_, and the virtual 2π_u_*, referred to as CAS(8,6), has been used, which will suffice to account for the largest part of nondynamical electron correlation contribution to the total energy of both, neutral, and core ionized states. For the organic molecules, a CAS containing the full space of molecular orbitals coming from the p-orbitals of C and O (or F) and the H(1s) has been considered. That means a CAS(8,8) for formaldehyde, a CAS(12,12) for formic acid, a CAS(10,10) for methanol, and the isoelectronic fluoromethane molecule. Smaller active spaces have also been considered by leaving out of the active space the molecular orbitals with occupations closer to two and zero, that is, CAS(6,6) for formaldehyde and a CAS(8,8) for both formic acid and methanol.

## Results and Discussion

We start the discussion focusing on the CO_2_, CS_2_, and COS molecules; all three share a linear structure and a C atom simultaneously involved in two double bonds, although with different charge separation as the electronegativity of O is larger than that of S, implying that, from a valence bond picture, the ionic forms will have larger contribution in the former molecule. Results in Table 1 show that as already observed for CO,^25^ the C(1s) CLBE in CO_2_ is larger than the experimental value by roughly 1.8 eV; this is significant as the MAE for ΔSCF-HF predicted CLBEs is below 0.5 eV. A similar situation is found for CS_2_ where the C(1s) CLBE is also larger than the experimental value by almost the same amount. On the other hand, the O(1s) CLBE in CO_2_ is ∼0.5 eV smaller than the experimental value, as expected. This is also the case for the O(1s) in COS, while for the S(2s) CLBE in the CS_2_ and COS molecules, the ΔSCF-HF almost matches the experimental value. All in all, the reported ΔSCF-HF values indicate the presence of strong nondynamical correlation effects, likely to be larger in CO_2_, as, here, one expects that the ionic valence bond resonant forms are thought to have a larger contribution. This is indeed supported by the calculated electron correlation contribution to the total energy which is −2.24, −2.14, and −1.85 eV for the neutral CO_2_, COS, and CS_2_ molecules, respectively, in all cases corresponding to the CASSCF calculation with the CAS(8,6) wave function. Results in Table 1 also show that CASPT2 values, including the effect of dynamic electron correlation, are closer to experiment, as expected, although the improvement with respect to CASSCF is quite small, indicating the dominant role of nondynamic correlation for the calculation of CLBEs.

**Table 1:** Core-Level BEs for the CO_2_, CS_2_, and COS Molecules Computed by ΔSCF-HF Calculations and CASSCF and CASPT2 Calculations with an Active Space of 8 Electrons and 6 Orbitals^a^

	ΔSCF-HF	CAS(8,6)	CASPT2	experiment^b^
**CO**_**2**_				
C(1s)	299.48	297.39	297.56	297.69
O(1s)	540.73	540.45	541.21	541.28
**CS**_**2**_				
C(1s)	294.9	293.2	292.9	293.1
S(2s)	234.2	234.1		234.2
**COS**				
C(1s)	297.3	295.3	295.1	295.2
O(1s)	539.8	539.9	540.0	540.3
S(2s)	235.0	234.6		235.0

a All values are given in eV. The number of significative figures in the calculated values is as in the experimental ones reported by Jolly et al. in ref (14).

b Data from ref (14).

To further prove that the interpretation above is indeed correct, we focus now on the CLBEs estimated from the ΔSCF-CASSCF calculation with the CAS(8,6) choice of the active space and active electrons. For CO_2_, the C(1s) CLBE now becomes 0.3 eV smaller than experiment and the O(1s) becomes even smaller—0.8 eV, whereas the ΔSCF-HF value was 0.5 eV lower than experiment. In a similar way, the ΔSCF-CASSCF value for C(1s) CLBE of CS_2_ and COS now becomes 0.1 eV larger than experiment and the S(2s) smaller by 0.1 and 0.4 eV for CS_2_ and COS, respectively. These results clearly demonstrate that the excessive deviation of the ΔSCF-HF calculated CLBEs, often larger than experiment, is due to the presence of strong nondynamical electron correlation effects. These are inherent to the formation of chemical bonds but used to be especially large in multiple polar bonds. At this point, one may wonder whether the remaining difference between calculated and experimental values is due to relativistic effects. The contribution of relativistic effects to the C(1s) and O(1s) CLBEs as predicted from HF–Dirac calculations is 0.13 and 0.45, as reported in previous work.^17^ Adding these values to the ΔSCF-CASSCF value for C(1s) and O(1s) CLBEs lets values be even closer to experiment. The case of S(2s) is intriguing because the relativistic contribution, computed here at the same level as in ref (17)., is significantly larger (1.35 eV), meaning that CASSCF may not be enough to recover all differential correlation. At the nonvariational CASPT2 level, adding the relativistic contribution to C(1s) for CO_2_ and CS_2_ matches the experimental value, whereas for O(1s) in CO_2_, this is slightly larger than experiment. Nevertheless, the important point here is that ΔSCF-HF values larger than experiment are indicative of strong nondynamic correlation. It is noted in passing that fully reproducing the experimental absolute CLBEs is delicate because of the interplay between electron correlation and relativistic effects.

The discussion above suggests that the phenomenon observed in the so far investigated molecules is general rather than an exception. It strongly indicates that molecules involving carbon–oxygen double bonds as in carbonyl and carboxyl groups are likely to involve nondynamical electron correlation effects that make the ΔSCF-HF calculated CLBEs larger than experiment. Results in Table 2 show that for formaldehyde, formic acid, methanol, and fluoromethane, the ΔSCF-HF calculated C(1s) CLBE is larger than experiment. However, the difference to experiment varies from 0.14 eV for methanol and 0.18 eV for fluoromethane to 1.03 eV for formic acid, with formaldehyde lying in between with a difference to experiment of 0.4 eV. The rather small values for methanol and fluoromethane are attributed to the existence of just a polar single bond between C and the hydroxyl or fluoro group; the case of formaldehyde can also be understood as this molecule features a double carbon–oxygen bond and the difference to experiment is smaller than in CO_2_, featuring two double bonds. Finally, formic acid features simultaneously a double C=O bond and a single C–OH bond which, assuming that the difference is due to the presence of nondynamical electron correlation effects, explains the observed trend. In the three molecules, the ΔSCF-HF calculated O(1s) CLBEs are smaller than experiment with the differences following the same trends as the C(1s) CLBEs. Thus, the largest difference (−1.34 eV) is for the O atom of the C=O double bond in formic acid and the smallest one (−0.47 eV) is for methanol, with, again, the case of formaldehyde lying in between the two extremes (−1.22 eV). Again, these differences are larger than the MAE for the ΔSCF-HF calculated CLBEs in a larger number of molecules,^17^ thus pointing to the existence of physical effects that are not taken into account.

**Table 2:** Core-Level BEs Computed by ΔSCF-HF and CASSCF Calculations with Different Active Spaces and CAS (Number of Electrons and Number of Orbitals)^a^

				experiment^b^
**H**_**2**_**CO**	ΔSCF-HF	CAS(6,6)	CAS(8,8)	
C(1s)	294.87	294.14	293.83	294.47
O(1s)	538.22	538.37	538.10	539.44
**HCOOH**	ΔSCF-HF	CAS(8,8)	CAS(12,12)	
C(1s)	296.83	295.79	295.31	295.80
O(1s) (C=O)	537.58	537.75	537.87	538.92
O(1s) (C–OH)	540.44	539.90	539.73	540.65
**H**_**3**_**COH**	ΔSCF-HF	CAS(8,8)	CAS(10,10)	
C(1s)	292.56	292.43	291.84	292.42
O(1s)	538.15	537.89	537.89	538.62
**CH**_**3**_**F**	ΔSCF-HF		CAS(10,10)	
C(1s)	293.78		293.30	293.6
F(1s)	691.24		691.45	692.4

a All values are given in eV.

b Data from ref (14).

Accounting for nondynamical electron correlation in formaldehyde, formic acid, methanol, and fluoromethane within the active spaces described in the previous section leads to calculated C(1s) CLBEs that are all smaller that the experimental value, as expected from the arguments in previous work^25^ and as discussed in the introduction section. The difference from experiment depends on the active space chosen but varies between 0.3 and 0.8 eV and, thus, within the MAE corresponding to the ΔSCF-HF calculated CLBEs. The effect of nondynamical electron correlation on the O(1s) CLBE of these molecules is also to reduce the ΔSCF-HF calculated value. The effect is quite large, leading to differences to experiment between 0.7 eV for methanol and 1.3 for formaldehyde. In this sense, including nondynamical electron correlation worsens the agreement of the O(1s) CLBE with respect to experiment. In fact, the error is now much closer to the ∼1 eV expected between a 1s^2^ and a 1s^1^ configuration.^38^ Accounting for nondynamical electron correlation leads to CLBEs that tend to be smaller than those predicted by the ΔSCF-HF approach. Therefore, it is possible that in some cases, the good agreement between the ΔSCF-HF calculated CLBEs and experiment arises from a fortunate error compensation between correlation within the 1s shell and between the valence and core–shells.

Before closing this section, we note that recent work by Cunha et al.^39^ reports 1s core-level binding energies for a series of third-row elements where some HF ΔSCF values are larger than experiment. The present results strongly suggest that this unphysical result is also due to nondynamic electron correlation effects, and the same is likely to be the case for the 2s and other core levels.

## Conclusions

The CLBEs predicted from total energy differences obtained from a variational calculation are expected to be smaller than the experimental values. This is certainly the case for the CLBEs predicted by the HF method. However, there are cases where the thus-calculated CLBEs are larger than experiment. This is the case for the C(1s) core level of the CO molecule discussed at length in a previous work,^25^ which also showed that this is because the triple bond in these molecules leads to strong nondynamical electron correlation effects which are different for the initial state and the *Z* + 1 ionic state. Including the nondynamical correlation in the π space leads to C(1s) CLBEs that are smaller than experiment because this introduces the appropriate weight of covalent and ionic valence bond resonating forms in the involved chemical bonds. In the present paper, we have shown that this effect is not restricted to CO and presented evidence that also appears in a series of molecules including CO_2_, CS_2_, formaldehyde, formic acid, methanol, and fluoromethane. In all these cases, the HF C(1s) is larger than experiment and including nondynamical correlation leads to values smaller than experiment. The effect on the O(1s) is also significant, and even if the HF O(1s) is smaller than experiment, including nondynamic electron correlation makes them even smaller; thus, they occur with a larger deviation to experiment but closer to the error expected when correlation within the core–shell is neglected. The rather accurate values for the HF O(1s) CLBE in these molecules are the results of a fortunate error cancelation between the core–core and core–valence correlation neglected in our CASSCF wave functions. It is noted in passing that this distinction between the different types of electron correlations is hardly achievable in the context of DFT.

The results in the present work have been obtained for a reduced number of molecules, but the observed trends are likely to occur in molecules involving C=O bonds; this is in aldehydes, ketones, organic acids, and esters and to a lesser extent in molecules with polar C–O bonds like alcohols or C–F bonds. The analysis in this work also illustrates the danger of focusing on absolute CLBEs only. In this sense, the theoretical prediction of CLBE shifts is more robust and physically meaningful.^8,9^

Finally, the present results can be used to investigate whether a given density functional leads to the right answer for the right reason; this was investigated for the CO molecule,^27^ but further work is needed to assess the capability of the existing functionals to introduce the physically meaningful nondynamical electron correlation in this type of rather simple molecule as this approach does not permit us to distinguish among the different types of electron correlation.
